# Analyzing Sensor-Based Time Series Data to Track Changes in Physical Activity during Inpatient Rehabilitation

**DOI:** 10.3390/s17102219

**Published:** 2017-09-27

**Authors:** Gina Sprint, Diane Cook, Douglas Weeks, Jordana Dahmen, Alyssa La Fleur

**Affiliations:** 1 Department of Computer Science, Gonzaga University, Spokane, WA 99202, USA; 2 School of Electrical Engineering and Computer Science, Washington State University, Pullman, WA 99163, USA; cook@eecs.wsu.edu; 3 St. Luke’s Rehabilitation Institute, Spokane, WA 99202, USA; weeksdl@st-lukes.org; 4 School of Biological Sciences, Washington State University, Pullman, WA 99163, USA; jordana.dahmen@wsu.edu; 5 Department of Mathematics and Computer Science, Whitworth University, Spokane, WA 99251, USA; alafleur19@my.whitworth.edu

**Keywords:** physical activity monitoring, wearable sensors, change detection, Fitbit, inpatient rehabilitation, pervasive computing

## Abstract

Time series data collected from sensors can be analyzed to monitor changes in physical activity as an individual makes a substantial lifestyle change, such as recovering from an injury or illness. In an inpatient rehabilitation setting, approaches to detect and explain changes in longitudinal physical activity data collected from wearable sensors can provide value as a monitoring, research, and motivating tool. We adapt and expand our Physical Activity Change Detection (PACD) approach to analyze changes in patient activity in such a setting. We use Fitbit Charge Heart Rate devices with two separate populations to continuously record data to evaluate PACD, nine participants in a hospitalized inpatient rehabilitation group and eight in a healthy control group. We apply PACD to minute-by-minute Fitbit data to quantify changes within and between the groups. The inpatient rehabilitation group exhibited greater variability in change throughout inpatient rehabilitation for both step count and heart rate, with the greatest change occurring at the end of the inpatient hospital stay, which exceeded day-to-day changes of the control group. Our additions to PACD support effective change analysis of wearable sensor data collected in an inpatient rehabilitation setting and provide insight to patients, clinicians, and researchers.

## 1. Introduction

In recent years, wrist-worn devices, such as pedometers and fitness trackers, have increased in popularity as people aim to be more physically active and aware of their overall health. One of the most popular consumer-grade physical activity monitors is the Fitbit. Fitbits are quite popular because of their ease of use and relatively low cost. Wrist-worn devices, such as the Fitbit, are able to provide rich information on user activity such as physical activity, heart rate, and sleep quality. Physical activity is estimated by fitness trackers and pedometers, such as the Fitbit, by measuring the steps taken by the wearer [1]. In addition to personal fitness, objective measures of physical activity are useful for healthcare purposes. For example, wrist-worn devices have been the subject of research in studies with aims ranging from assessing the daily walking structures of post-stroke adults [2] to qualitatively evaluating the physical activity of children in rehabilitation [3].

In inpatient rehabilitation, clinical observations by therapists are typically used to characterize patient progress and make treatment decisions. It can be beneficial for physical therapists to obtain objective physical activity information for patients during their stay in inpatient rehabilitation. There are several benefits of sensor-based physical activity measurements, including overcoming the inaccuracy of self-report by patients. Patients often do not remember their activities or have a biased interpretation of how well they are doing, consequently leading to either an over or under estimation of daily activity. In comparison to human observation alone, wrist-worn devices are able to objectively perform continuous tracking of physical activity, thus reducing the subjectivity introduced by human observation. Finally, wrist-worn devices, such as the Fitbit, can gather fine-grained physical activity data about patients that physical therapists cannot otherwise observe.

Several studies and review articles in this growing area of research include a call for both further studies on the efficacy of these devices [4], their uses in a clinical setting [3,5,6], and convenient and efficient ways to analyze the data generated by their use [7]. In this study, we investigate physical activity and changes in physical activity of individuals undergoing inpatient rehabilitation using wrist-worn fitness trackers. More specifically, we detect and analyze changes in fine-grained, continuous physical activity and heart rate data collected from Fitbits worn by individuals in two groups: REHAB and CONTROL. The REHAB group consisted of patients undergoing inpatient rehabilitation at a large inpatient rehabilitation facility. To provide a comparison for the physical activity changes exhibited by rehabilitation patients over the course of recovery, we also collected continuous physical activity data from a healthy population, the CONTROL group. To detect and analyze changes in both populations we use the Physical Activity Change Detection (PACD) framework from our earlier work [8,9,10]. PACD is a framework that windows sensor-based time series data into time periods, detects changes between time periods, computes the significance of the detected changes, and examines the source of the changes. In this paper, we expand PACD to analyze the longitudinal physical activity data collected from both groups to gain insights into the detected changes over time in both an inpatient setting and a free-living setting. As part of our analysis, we introduce unsupervised change detection and analysis methods to investigate change within the groups, change between the groups, and change within the individual. The results of our analysis provide insight into the recovery process for inpatient therapy populations, as well as a framework for detecting change that can be applied to domains outside of inpatient rehabilitation.

## 2. Related Work

Previous research has investigated sensor-based physical activity monitoring for inpatient rehabilitation [5,6,11]. The Stroke Inpatient Rehabilitation Reinforcement of ACTivity (SIRRACT) trial was the first international, multi-facility trial to deploy wearable sensors for patients undergoing stroke rehabilitation [5]. Data were collected from tri-axial accelerometers worn on each ankle from 135 participants in 11 different countries. Therapists fit the sensors on participants in the morning and removed the sensors in the evening. From the acceleration signals, walking bouts were identified. Metrics related to walking bouts were computed, including: speed, duration, number of walking bouts, average walking speed, total time walking, total distance, and total steps taken. The participants were split into two groups: one only receiving feedback regarding their walking speed and one receiving, in addition to walking speed information, feedback in the form of activity graphs. The results indicated no significant differences between the two feedback groups in daily time spent walking (15.1 ± 13.1 min for walking speed feedback only compared to 16.6 ± 14.3 min for activity graph feedback). Additionally, 30% of participants decreased their total daily walking time over the course of inpatient rehabilitation and the majority of walking bouts only lasted between 10 and 30 s [5]. More recent studies investigating walking bout feedback during inpatient rehabilitation are published by Hornby and colleagues [6] and Mansfield and colleagues [11].

In regards to change detection approaches, a handful of studies have investigated change that can be detected in human behavior patterns. These approaches have quantified change statistically [12,13,14], graphically [13,15,16], and algorithmically [15,17,18,19]. Recently, Merilahti et al. [12] extracted actigraphy-based features from data collected for at least one year. The authors individually correlated each feature with a component of the Resident Assessment Instrument [20] to gain insights into the relationship of longitudinal changes in functioning and actigraphy. While this approach provides information about the relationship between clinical assessment scores and wearable sensor data, this study does not directly quantify sensor-based change. Another activity-based change detection approach was proposed by Wang et al. [15]. In this study, passive infrared motion sensor data were analyzed to estimate physical activity in the home and also to estimate time away from home. The data were converted into co-occurrence matrices for the computation of image-based texture features and visualization with activity density maps. Wang and colleagues’ case studies suggest the proposed texture method can detect lifestyle changes, such as knee replacement surgery and recovery. More recently, Tan et al. [16] applied the texture method of Wang and colleagues to sensor data collected from Fitbit Flex sensors. The goal of this study was to detect and track changes in daily activity patterns for elderly participants. Furthermore, smart-home data has been analyzed to track mobility and time away from home with regards to physical and cognitive health [15,21].

In one of our earlier works, we demonstrated that passively-collected, longitudinal smart-home data can be analyzed to predict performance on cognitive health-assessment tests [17,22] and detect major health events [9,10]. We previously proposed the Physical Activity Change Detection (PACD) framework for investigating changes in physical activity data collected from Fitbit devices [8]. With PACD, we analyzed 12 days of Fitbit Flex data collected from 11 individuals in free living situations. For each individual, the data were segmented into two windows of six days. We performed a single comparison between the two windows around a candidate change point. In this paper, we expand PACD to perform multiple comparisons on window sizes that are daily windows in order to handle data collection periods that are not uniform in length across participants, and to investigate changes within and between groups of participants.

## 3. Methods

Physical activity (PA) is often defined as any bodily movement by skeletal muscles that results in caloric energy expenditure [23]. PA is divided into bouts of physical movement and periods of physical rest. Furthermore, PA bouts are described and quantified by four components:

1. Frequency: the number of bouts of physical activity within a time period, such as a day.
2. Duration: the length of time an individual participates in a single bout.
3. Intensity: the physiological effort associated with a particular type of physical activity bout.
4. Activity type: the kind of exercise performed during the bout.

To add exercise throughout the day, a person can increase their number of bouts (frequency), increase the length of bouts (duration), increase the intensity of bouts (intensity), and vary the type of physical activity performed during the bouts (activity type). These four dimensions of physical activity correspond to four distinct types of changes that represent progress toward many different health goals, such as increasing physical activity or improving consistency in one’s daily routine. In this paper, we study the problem of detecting and analyzing change in PA patterns using these four dimensions. More specifically, we utilize algorithmic approaches to determine if a significant change exists between two windows of time series step data sampled from a PA sensor device, such as a Fitbit. The following sections describe the participants from which PA data were collected, the wrist-worn physical activity device employed, pre-processing applied to the data for cleaning purposes, the PACD method for change detection and analysis, and features extracted from the continuous PA data.

### 3.1. Participants

To gain insights into physical activity levels during inpatient rehabilitation, we compared two subject groups, a REHAB (inpatient rehabilitation patients) group and a CONTROL (healthy adults) group. Participants in the REHAB group were recruited from the inpatient population of a large medical rehabilitation facility. A regional hospital institutional review board approved the study and all participants gave written informed consent before they participated in the study. In the United States, patients in inpatient rehabilitation facilities are required to obtain a minimum of 3 h of therapy a day. This can be any combination of physical, occupational, or speech therapy with the therapy regimen tailored to the impairments/needs of the patient. The ratio of different types of therapy can change from day to day depending on improvement throughout the stay. For the REHAB group, patients were recruited by therapist recommendations based on the following criteria: mobile-capable, older than 18 years of age, English speaking, recently admitted, and appearing cognitively capable for the study, as measured by a Mini-Cog exam (score > 0 for participation) [24]. Fifteen participants (Male = 8, Female = 7) between 44 and 80 years of age (61.33 ± 12.66 years of age) participated in the study during the duration of their inpatient rehabilitation stay. To assess participants’ physical activity two weeks prior to their hospitalization, an International Physical Activity Questionnaire for the Elderly (IPAQ-E) [25] was administered on the second day of participation.

**Figure 1:**
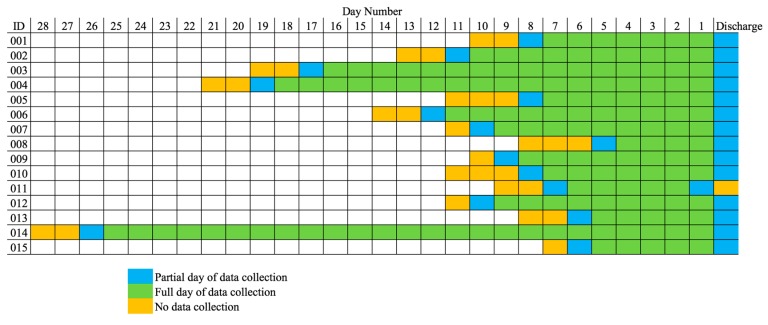
Framing of when data collection occurred during each REHAB participant’s inpatient rehabilitation stay.

Table 1 shows individual participant characteristics, including each participant’s admission and discharge Functional Independence Measure (FIM) motor score. The FIM assessment is administered by clinicians to determine independence in activities of daily living (ADLs) [26]. The FIM is a well-validated assessment that measures a patient’s functional abilities on a 0–7 rating scale for 18 items. The 18 items are designed to represent six motor and cognitive domains: self-care, sphincter control, transfers, locomotion, communication, and social cognition. We include the sum of the scores for the motor function items of the FIM. Fitbit data collection began as soon as a participant was recruited to the study, which was 1.93 ± 0.70 days after admission. For all participants, except 011, data collection ended on the same day as discharge. Not counting partial days at the beginning and end of data collection, the number of full days they participated in the study ranged from four to 25 days (9.73 ± 5.79 days). Figure 1 is a visualization of how each REHAB participant’s data collection period is framed in their total length of stay.

The CONTROL group consisted of nine healthy participants (Male = 4, Female = 5) between ages 18 and 25 (21.22 ± 2.22 years of age) recruited from the general population in a university town. These participants were selected based on the following criteria: ability to receive text messages, willingness to wear Fitbits continuously for a two-week period, 18+ years of age, and in healthy condition. Participants in the CONTROL group were administered an IPAQ short form questionnaire to assess their physical activity prior to the study.

### 3.2. Instrumentation

Participants in both groups wore Fitbit Charge Heart Rate (HR) monitors on their wrists. The FitBit Charge HR activity tracker uses a wrist-mounted three-axis accelerometer to detect intensity of movement. A wrist-worn activity tracker was chosen over a waist-mounted tracker to be able to measure heart rate and sleep characteristics, and to reduce the risk of loss of the tracker as patients transfer from supine lying to sitting to standing, and vice versa, many times during the inpatient rehabilitation day. The devices were placed on the wrist associated with the non-dominant arm and we asked participants to wear the Fitbits at all times. The activity tracker was worn day and night in order to quantify step count and heart rate per 24 h period.

For the REHAB group, we set up two Charge Heart Rate (HR) Fitbits for each patient, with one being attached and the other acting as a fully-charged alternate. The participants wore the devices at all times during inpatient treatment. We administered daily check-ins to make sure the device was being worn and if it had been taken off for any reason. We also checked the skin integrity of patients and the Fitbits for adequate battery charge. Every four days, Fitbits were swapped for syncing data to the Fitbit servers, disinfecting, and charging.

For the CONTROL group, we provided participants with a single Fitbit Charge HR. Participants were provided with instructions for how to properly wear and sync the device. We sent daily text messages to remind participants to wear and sync the devices. Text instruction were also given when low battery status alerts were sent to the investigators from Fitbit to charge the Fitbit that night during sleep, and then resume wear the following day. Fitbits were returned after the two weeks of data collection.

Since we employed the Fitbit Charge HR in a rehabilitation setting, research investigating the accuracy of Fitbit step counts and heart rate for adults with physical impairments are highly relevant. A wrist-worn FitBit has been shown to not differ significantly in quantifying step count per minute and over a 24-h period when compared to actigraphy during light, moderate, and vigorous intensity physical activity in healthy adults in free-living conditions [27]. In a cardiac rehabilitation population, step counts from a wrist-worn FitBit were shown to correlate with step count estimates from an Actigraph (*r* = 0.95), although it was reported that the FitBit tended to over-count steps [28]. By contrast, in older adults with impaired ambulation, a wrist-worn FitBit was reported to underestimate step count by 16% compared to direct observation of steps during a self-paced walking test [29]. Finally, in patients with relapsing and progressive multiple sclerosis with a broad range of ambulatory disability, step counts from a wrist-worn FitBit correlated 0.69 with direct observation of steps during a 2-min walk test, while the direct observation-Actigraph correlation was 0.76 [30]. In a recent study, step counts from seven activity monitors, including a Fitbit charge (no heart rate), were collected from 166 rehabilitation patients. When compared with direct observation by therapist, the Fitbit Charge worn on the wrist had the fourth highest intraclass correlation (ICC) (0.399), while a StepWatch worn on the ankle had the highest ICC (0.982) and an Actigraph worn on the hip had the lowest ICC (0.123). Overall, validity for step counts from wrist-worn Fitbits relative to direct observation and the Actigraph is reported to be moderate to excellent, with intensity of motion necessary to count a step being relatively stable within a person. Moderately strong correlations between the heart rate measurement from the FitBit Charge HR and electrocardiogram, as the gold standard, have been obtained in healthy adults during various degrees of physical effort from rest to vigorous activity (*r* range 0.81–0.83) [31,32]. The FitBit Charge HR has been reported to underestimate heart rate by a small margin (3.4%) compared to electrocardiogram during slow to moderate speed walking (2 km/h and 3.5 km/h) in healthy adults [33]. We were unable to locate literature reporting on accuracy of the FitBit Charge HR for quantifying heart rate in patient populations.

### 3.3. Data Pre-Processing

We downloaded daily summary and minute-by-minute physical activity (as measured by step count) and heart rate data collected from Fitbit Charge HR devices via the Fitbit application programming interface (API). Pre-processing of the downloaded data includes handling missing data. We handled missing data by restricting the data analysis to complete 24-h days of data collection. To do this, the first and last days of data collection are considered partial days, including the evening on the first day and the morning on the last day. We exclude these partial days from our analysis since they are not full days; however, for some features that make use of previous night or next morning information, we do use relevant data from the partial days. Next, we consider data collection days that do not contain any steps taken during 7:00 A.M.–9:00 P.M. to be missing data. Zero steps during the day is likely due to removing the Fitbit to charge it, then forgetting to put it back on until much later. For the REHAB group, only two days are considered missing and were removed from analysis. For the CONTROL group, only one day is considered missing and was removed from analysis. Finally, there are some minutes within a day that have missing heart rate data, possibly due to improper placement on the wrist or the device being removed to shower or wash hands. If the period of missing of heart rate data is less than, or equal to 30 min, the heart rate data is interpolated between the two heart rate values surrounding the missing data period. If the period of missing heart rate data is greater than 30 min, we assume the device is doffed and we fill the heart rate data with the resting heart rate for the day in question, which is measured by Fitbit and provided for a specific date via the Fitbit API. We fill missing heart rate data with resting heart rate to ensure we underestimate physical activity rather than overestimate.

### 3.4. Physical Activity Change Detection

Originally, we designed PACD to detect and analyze changes for an individual from his/her PA time series data. In addition to individual-level analysis, in this paper we are interested in detecting and analyzing PA changes exhibited by groups, specifically the REHAB group and the CONTROL group. To do this, we introduce expansions to PACD to support group analysis. Before presenting these additions, we will first provide an overview of PACD and its associated change score algorithms that we have used for individual analysis.

**Figure 2:**
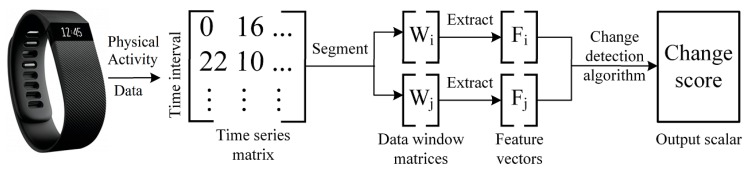
Overview of the Physical Activity Change Detection (PACD) framework.

PACD quantifies the magnitude of change in PA patterns between two segments of sensor-based time series activity data that were sampled by a wearable fitness device or a pedometer, such as a Fitbit. PACD is a framework that windows sensor-based time series data into time periods, detects changes between time periods, computes the significance of the detected changes, and examines the source of the changes. Figure 2 illustrates the first three steps of the PACD process. Let X denote a sample of sensor-based time series data that has been divided into days, $X=\left\{D_{1},D_{2},\ldots \right\}$ where each day consists of minute-by-minute vectors of PA data x, $D=\left\{x_{1},x_{2},\ldots x_{1440}\right\}$. Since there are 1440 min per day, there are 1440 PA data vectors collected per day D. For the current study, x represents a single minute of step and heart rate values. Let W be a window of n days such that W⊆X. PACD compares two windows of data, $W_{i}$ and $W_{j}$, within time series X. Additionally, $\hat{W}$ is an aggregate window that corresponds to the average of all days within the window W:

(1) $$\hat{W}=\frac{1}{n}\overset{n}{\underset{i=1}{\sum }}D_{i},D_{i}\in W$$

Once the data has been windowed (and possibly aggregated, depending on the analysis being performed), two windows $W_{i}$ and $W_{j}$ are compared in one of two window modes: baseline or sliding. To denote a comparison between two windows $W_{i}$ and $W_{j}$, we use the notation $W_{i}\Delta W_{j}$. For a baseline window comparison, the first window (i=1) of data is considered a reference window that is sampled from the beginning of the time series. This reference window, $W_{1},$ is included in each comparison. Beyond the reference window, the remaining windows (j=2,3,…) are compared to the baseline window, $W_{1}$. For a sliding window comparison, both windows used for comparison are advanced through the time series data, one window in front of the other (i=1,2,… and j=2,3,…). To detect change between two windows, PACD can make use of any function that accepts two windows as input, $W_{i}$ and $W_{j}$, and outputs a scalar change score, CS, that represents the change between $W_{i}$ and $W_{j}$. A higher value of CS indicates a higher amount of detected change between the two windows. Once a change score has been computed, the next step is to perform significance testing of the change score, *CS*. This step is necessary to interpret the change score’s magnitude. If a change score is significant, as determined by a significance test, the next step in PACD is to determine the nature and the source of change. Typically, this step requires the computation of features that summarize the data and provide a meaningful context for change. For example, the total number of steps taken per day is one such PA feature that is simple and commonly known. The change between daily steps from one window to the next window can be computed and utilized as an explanation of change. There are several methods to capture change across time in individual features. A common approach is to compute the percent change for a feature f from a previous window $W_{i}$ to a current window $W_{j}$:

(2) $$f_{\Delta \%}=\frac{f_{W_{j}}-f_{W_{i}}}{f_{W_{i}}}\times 100$$

Two sample tests or effect size analyses are two examples of statistical methods that can also be applied to quantify change between two samples; however, the multiple testing problem should be accounted for with a method such as the Bonferroni or Benjamini-Hochberg correction [20]. In our own earlier work, we explored using two sample tests to quantify the change between two windows of Fitbit-collected step and heart rate data [14].

### 3.5. Change Score Algorithms

In our previous work, we evaluated several change score functions and their applicability to different types of data. For this paper, we utilize three change detection methods: small window adaptation of the Permutation-based Change Detection in Activity Routine algorithm (sw-PCAR), distance-based dissimilarity scorer (DDS), and virtual classifier (VC). Using different change detection methods provides a diverse set of perspectives on the data comparison. For example, DDS and VC compute change scores based on features extracted from each window. On the other hand, sw-PCAR does not utilize window features and computes a change score directly from the time series data. It is beneficial to compute the results of several change score algorithms because if more methods detect a significant change, then there is greater evidence detecting a notable change in PA.

#### 3.5.1. Method 1: sw-PCAR

We originally designed the Permutation-based Change Detection in Activity Routine (PCAR) approach [17] to analyze changes in longitudinal smart-home data without the need for a feature extraction step. We adapted the original implementation of PCAR to handle smaller windows of activity-labeled data, yielding the small-window PCAR (sw-PCAR) algorithm. sw-PCAR is a data-driven approach that computes a change score based on the minute-by-minute PA data, not based on features. For sw-PCAR, we average the days within two windows $W_{i}$ and $W_{j}$ using Equation (1) to yield aggregate windows $\hat{W_{i}}$ and $\hat{W_{j}}$. Next, we compute CS using the symmetric Kullback-Leibler (KL) divergence distance between the minute-by-minute PA vectors in $\hat{W_{i}}$ and $\hat{W_{j}}$. Finally, we compute the significance of the distance value CS by concatenating data from windows $\hat{W_{i}}$ and $\hat{W_{j}}$ into one window W. All the time intervals within W are randomly shuffled and then split into two new sub-windows. Lastly, we compute the KL distance for this permuted window pair.

To produce an N-length vector V of KL distances, we repeat this shuffling procedure N times. If N is large enough, the corresponding set of KL distances forms an empirical distribution of the possible permutations of activity data for the two windows. For sw-PCAR, we determine change significance using an approach proposed in our earlier work [8]. For this test, we compare CS to the permutation vector V with boxplot-based outlier detection. For this method, the interquartile range, which corresponds to the 75th percentile–25th percentile, of V is computed. Values outside of the 75th percentile + 1.5 × interquartile range are considered outliers [34]. If CS is determined to be an outlier of V, then we conclude the change score is significant.

#### 3.5.2. Method 2: Distance-based Dissimilarity Scorer

We propose a simple, daily change score algorithm, Distance-based Dissimilarity Scorer (DDS), that computes a change score based on dissimilarity between features. First, a feature extraction step reduces two windows $W_{i}$ and $W_{j}$ into two n×z feature matrices, $F_{i}$ and $F_{j}$, where n is the window size (in days) and z is the number of features that are extracted (see Section 3.7 for feature descriptions and Table 2 for a description of the z features). Example features extracted include the total steps taken and the number of bouts in different heart rate zones. For DDS, each feature matrix F is then aggregated into a feature vector $\hat{F}$ by averaging each feature value across days (similar to the computation of $\hat{W}$ in Equation (1)). A weighted normalized Euclidean distance (WNED) measure is used to produce a change score, CS. This change score quantifies the differences between the corresponding feature vectors $\hat{F_{i}}$ and $\hat{F_{j}}$. The smaller the WNED distance between these two vectors, the more similar the two days of data are. For WNED, each feature is normalized as follows [15]:

(3) $${\hat{F}}_{i}^{*}=\frac{\hat{F_{i}}\left(k\right)}{max\left[\hat{F_{i}}\left(k\right),\hat{F_{j}}\left(k\right)\;\right]}$$

(4) $${\hat{F}}_{j}^{*}=\frac{\hat{F_{j}}\left(k\right)}{max\left[\hat{F_{i}}\left(k\right),\hat{F_{j}}\left(k\right)\;\right]}$$

For k=1,…,z. Then, the WNED between $F_{i}^{*}$ and $F_{j}^{*}$, $d_{ij}$, is defined as follows:

(5) $$d_{ij}=\sqrt{\overset{d}{\underset{k=1}{\sum }}\frac{1}{d}{\left[{\hat{F}}_{i}^{*}\left(k\right)-{\hat{F}}_{j}^{*}\left(k\right)\right]}^{2}}$$

In the current DDS implementation, each feature is weighted equally. In the future, some features may be weighted more heavily on an individual participant basis. To determine the significance of the change between two windows using the DDS approach, we adopt a significance cut-off value of 0.25 that was used by Wang and colleagues for texture-based dissimilarity [15].

#### 3.5.3. Method 3: Virtual Classifier

The last method is called the virtual classifier (VC) approach. VC makes use of a binary classifier to detect and explain behavior change. This type of virtual classifier for change analysis was first proposed by Hido and colleagues [19]. For this approach, each feature vector in the feature matrix $F_{i}$, corresponding to window $W_{i}$, are labeled with a positive class and each feature vector in the feature matrix $F_{j}$, corresponding to window $W_{j}$, are labeled with a negative class (see Section 3.7 for feature descriptions). Using k-fold cross-validation (k=5), VC trains a decision tree to distinguish between the virtual positive and negative classes. The resulting average prediction accuracy is represented as $p_{VC}$. If a significant change exists between $W_{i}$ and $W_{j}$, the average classification accuracy $p_{VC}$ of the learner should be higher than the accuracy expected from random noise: $p_{rand}=0.5$, the binomial maximum likelihood of two equal-length windows.

For the VC approach, we used the inverse survival function of a binomial distribution to determine a critical value, $p_{critical}$, at which n Bernoulli trials were expected to exceed $p_{rand}$ at α=0.05 significance. If $p_{VC}\geq p_{critical}$, a significant change exists between windows $W_{i}$ and $W_{j}$. After a significant change has been detected, VC provides an explanation of the source of change without reliance on statistical tests. Upon significant change detection, retraining a decision tree and inspecting the tree reveals which features are discriminatory in learning the differences between two windows. This explanation of change is one of the advantages of the VC approach over other change point detection algorithms.

For individual participant analysis, the VC approach is useful for large window sizes because each daily feature vector is treated as if it were a sample. In the REHAB group, participants have as few as four days of full data collected (FDC). Consequently, we only use the VC approach individual analysis for participants with FDC ≥10 days. In the following section, we will discuss how we use the VC approach for group analysis.

### 3.6. PACD for Group Analysis

For the REHAB group, the number of full days of data collection, FDC, is dependent upon when the participant was recruited to the study and their length of stay (LOS). While LOS is dependent on a patient’s recovery and suitability for discharge based on therapists’ expertise, LOS is also affected by factors unrelated to recovery, such as days allowed by insurance. For these reasons, the number of days available for data analysis is varied across the participants. In order to compare results amongst participants and summarize the changes exhibited by the REHAB group, normalization of FDC is necessary. To do this, we split each participant’s data into a fixed number of bins. We first identified three groups of participants who had a minimum FDC, S:

1. S=4 (FDC ≥ 4 days): N = 15 (all participants)
2. S=7 (FDC ≥ 7 days): N = 11 (including 001, 002, 003, 004, 005, 006, 007, 009, 010, 012, 014)
3. S=10 (FDC ≥ 10 days): N = 5 (including 002, 003, 004, 006, 014)

For each value of S (4, 7, 10), we split each participant’s daily data into S near-equal bins B. For example, participant 001 has FDC = 7. The resulting bins for a split of S=4 would be like the following:

- $B_{1}$: $D_{1},D_{2}$
- $B_{2}$: $D_{3},D_{4}$
- $B_{3}$: $D_{5},D_{6}$
- $B_{4}$: $D_{7}$

For the days in each bin, $B_{i}$, we compute an aggregate window, ${\hat{W}}_{i}$, to normalize the FDC time periods across participants. All aggregate windows ${\hat{W}}_{i}$ sorted in original, chronological order form a new aggregate time series $\hat{X}$. For the sw-PCAR and DDS approaches, PACD is applied to $\hat{X}$ with a window size of n=1 bin (one aggregate day). For each bin, all participant’s sw-PCAR and DDS scores are averaged to represent the group change. Adapting the VC approach for group analysis is different from the adaptations of sw-PCAR and DDS. To use VC for group analysis, each bin $B_{i}$ contains N feature vectors, one for each participant measured at $B_{i}n_{i}$. For example, when S=4 and we are performing a comparison between $B_{1}\Delta B_{2}$, there are 30 total feature vectors (15 participants, each with two feature vectors, one extracted at $B_{1}$ and one extracted at $B_{2}$).

Another PACD enhancement for group analysis is the inclusion of reference data. With the CONTROL group dataset, we have estimates of natural PA variability expected for a healthy population. We can leverage this information as normative values to help measure significance of the detected changes. If changes detected in the REHAB group are greater than the changes detected in the CONTROL group, we can conclude the REHAB changes are greater than expected from natural variability. Additionally, for individual features, CONTROL feature values can be used to label a direction of REHAB change as an improvement or regression. If the trend of a feature extracted from the REHAB group approaches the distribution of the same feature extracted from the CONTROL group, we can conclude that an improvement in the REHAB population is observed.

### 3.7. Feature Extraction

We extract features from the PA data (see Table 2) for two primary purposes: (1) to provide input features for the DDS and VC approaches (sw-PCAR does not utilize features for change detection) and (2) to provide an explanation of changes discovered by change detection algorithms (see Section 3.5). For this study, all features extracted are on a daily basis, D, meaning features that require multiple days are not included in the analysis. For each day $D=\left\{x_{1},x_{2},\ldots x_{1440}\right\}$ in a window of n days, we extract z features to produce a n×z feature matrix F. We group features in F together based on the period of the day used for computation: (1) daytime (7:00 A.M.–9:00 P.M.) or (2) nighttime (9:01 P.M. on the previous day to 6:59 A.M. on the current day). Nighttime can be further broken into the following two periods: (1) nighttime-morning (12:00 A.M.–6:59 A.M.), or (2) nighttime-evening (9:01 P.M.–11:59 P.M.). The cutoff values for daytime and nighttime correspond to the natural rhythms of an inpatient setting and were selected in collaboration with therapists. Daily features include PA summaries based on frequency, duration, intensity, and variability of PA bouts, as measured by step counts and heart rate.

For step count-based measures, we compute bouts of daily PA based on step count by using a threshold, ε. We consider sequences of time series data with steps greater than ε to be a bout of PA. If ground truth activity labels, such as walking, commuting, and biking are available from the device user and/or an activity recognition algorithm [22], PA type can be inferred and ε can be updated dynamically based on which activity the device user is performing. For this study, we assume such labeled information is not available and set ε=5 steps. By setting this value to 5 we are assuming physical activity is characterized by at least five steps per minute.

For heart rate-based measures, we take a similar approach; however, we define our PA measures to align with standards for therapeutic aerobic benefit. For each participant, we compute their maximal heart rate ($HR_{max}$) using age-estimated formulas from the literature. For patients not taking beta-adrenergic blockade (BB) medications, $HR_{max}$ is calculated as: $HR_{maxBB}=212-0.7\times age$ [35]. For patients taking beta-adrenergic blockade medications, $HR_{max}$ is calculated as $HR_{maxBB}=164-0.7\times age$ [36]. Using $HR_{max}$, PA bout intensity is considered to be in the target HR (THR) zone sufficient to obtain cardiovascular benefit when the 1-min HR value was 55% to 80% of $HR_{max}$. The HR response is considered to be excessive if the HR bin value exceeded 80% of $HR_{max}$. Duration of HR within the THR zone is quantified as number of consecutive minutes of HR within the zone and duration of excessive HR (EHR) is quantified as number of consecutive minutes of HR above the THR zone. Lastly, the frequency of HR bouts in the THR of at least 10 min duration are summed per day and the frequency of HR bouts in excess of the THR, regardless of duration, are summed per day.

## 4. Results

To investigate if PACD can detect and quantify physical activity changes during inpatient rehabilitation, two datasets are analyzed, REHAB and CONTROL. The REHAB dataset comprises 15 participants undergoing inpatient rehabilitation and the CONTROL group comprises nine healthy volunteers. To summarize the REHAB group, most participants were recovering from a stroke (eight participants) and rated themselves as moderately (three participants) to highly active (eight participants) pre-hospital as determined by the IPAQ-E. The participants’ length of stay at the inpatient rehabilitation hospital was 12.73 ± 5.74 days. Prior to serving as input to PACD, REHAB and CONTROL data are subject to pre-processing (see Section 3.3). In addition to the pre-processing described in Section 3.3, add-one smoothing is applied to avoid division by zero during sw-PCAR KL divergence computations. For PACD computations, the following algorithm parameter values are used: window mode = baseline; number of sw-PCAR permutations = 1000; VC cross-validation folds k=5. We tested the normalized window size S with values of S={4,7,10 days}.

**Figure 3:**
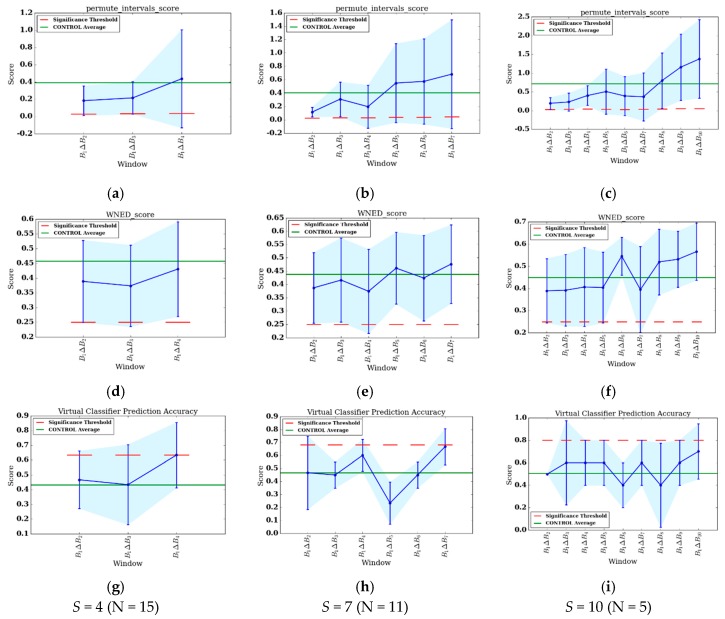
Change score results with standard deviation shown as error bars. (**a**–**c**) sw-PCAR; (**d**–**f**) DDS; and (**g**–**i**) VC; (**a**,**d**,**g**) S=4; (**b**,**e**,**h**) S=7; (**c**,**f**,**i**) S=10.

**Figure 4:**
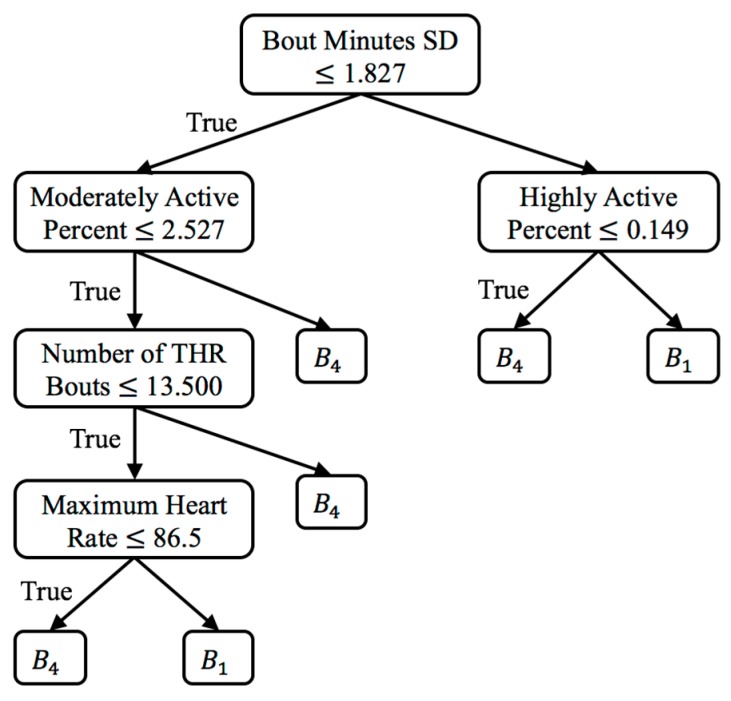
S=4 $B_{1}\Delta B_{4}$ VC top-level decision tree rules for change analysis. SD = standard deviation.

**Figure 5:**
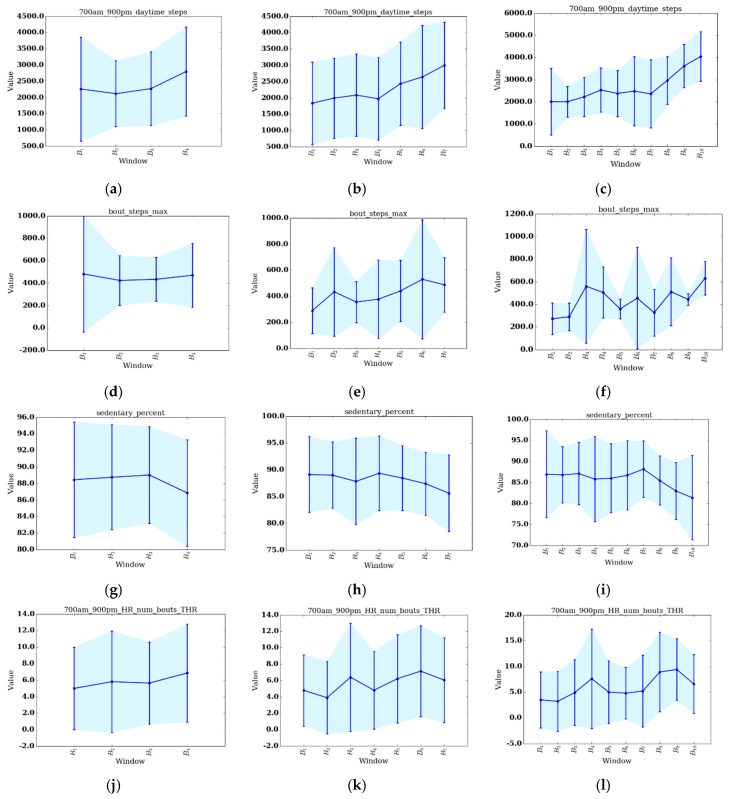
Average bin values for selected features with standard deviation shown as error bars. (**a**–**c**) daytime steps; (**d**–**f**) maximum number of steps taken in a bout during daytime; (**g**–**i**) sedentary percent; and (**j**–**l**) number of daytime bouts in the THR zone. (**a**,**d**,**g**,**j**) S=4; (**b**,**e**,**h**,**k**) S=7; (**c**,**f**,**i**,**l**) S=10.

To summarize group changes, Table 3 includes CONTROL and REHAB averages for each value of S and for each change score algorithm. Figure 3a–i show the change score curves for each value of S and for each change score algorithm, as well as the change significance threshold (red dashed line) and average CONTROL group score (green line). To provide context for the change scores and to perform change analysis, figures showing individual feature change are included. For the one significant VC result (S=4, CS=0.633), the top-level rules of the associated decision tree are shown in Figure 4. Plots of select features for each value of *S* are shown in Figure 5a–l. The selected features include daytime steps, maximum number of steps taken in a bout during daytime, sedentary percent, and number of daytime bouts in the THR zone. We selected these features for change analysis because of their clinical relevancy, trend, and ease of interpretation. To further support change analysis, we include individual REHAB participant S=4 values for the features of daytime steps (see Table 4) and number of daytime THR bouts (see Table 5). We hypothesize that using each of the change detection methods (sw-PCAR, DDS, and VC), PACD will detect PA changes (bout frequency, duration, intensity, and variability) for both groups, REHAB and CONTROL. However, we anticipate that the significance of the change will be much higher for the REHAB group, with greater change exhibited towards the end of the FDC period.

**Figure 6:**
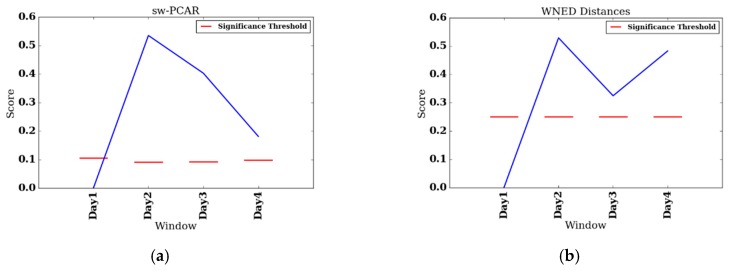
Daily change results for participant 008. (**a**) sw-PCAR; (**b**) DDS.

**Figure 7:**
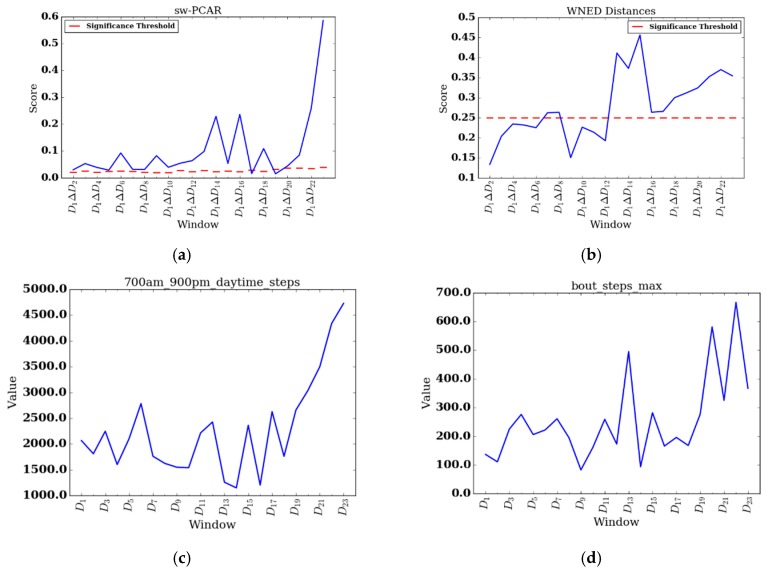
Daily change results for participant 014. (**a**) sw-PCAR; (**b**) DDS; (**c**) daily daytime steps; and (**d**) daily maximum number of steps taken in a bout during daytime.

To perform individual analyes of the REHAB group, we investigate two participants, participant 008, who had the shortest FDC (four days), and participant 014, who had the longest FDC (25 days). We also include change score plots for sw-PCAR and DDS for these two participants. Figure 6a,b show results for participant 008 and Figure 7a,b show results for participant 014. Furthermore, for participant 014 we include daily feature plots for daytime steps (see Figure 7c) and maximum number of steps taken in a bout during daytime (see Figure 7d). Parameters used for individual PACD analysis are the same as for group analysis, except the windows are not aggregated and the window size is one day (n=1).

## 5. Discussion

This paper presents our investigation of PACD for unsupervised change analysis of sensor-based PA and heart rate time series data. Three change detection algorithms are applied to two original datasets: (1) the CONTROL dataset, comprised of nine healthy participants’ Fitbit data and (2) the REHAB dataset, comprised of 15 inpatient rehabilitation participants’ Fitbit data.

### 5.1. CONTROL Group

The CONTROL group provides a reference of comparison for the REHAB group. Since the data were collected from healthy younger adults in their natural, free-living situations, we do not expect there to be any significant changes or trends. Using PACD, we have objective evidence to support this hypothesis (see Table 3). For the VC approach, the accuracy is as good as random guess ($p_{rand}=0.5$) and for all values of S there is not a baseline comparison that exceeds the significance threshold ($p_{critical}=0.667$). For the DDS approach, the change scores are 0.457, 0.434, and 0.449 for values of S=4,7,10 respectively, which is higher than the significance threshold of 0.25 (see Section 3.5.2). This suggests the DDS approach could benefit from a data-driven significance threshold approach. sw-PCAR results for S=4,7 are consistent (CS ≈ 0.4); however, there is a large spike for S=10 (CS=0.715). Since days within a bin are averaged to form an aggregate day, the variability for S=4,7 is washing out. For S=10 there are bins with only one day, so no aggregation is being performed. In summary, the CONTROL change scores for each of the three algorithms represent the natural day-to-day and bin-to-bin change we can expect from healthy younger adults.

### 5.2. REHAB Group

There are many observations that can be made from the REHAB PACD results. First, there are commonalities in the results for all values of S and for all three algorithms. Each comparison’s results are highly variable, as evident by the error bars in Figure 3a–i. Next, the last bin has the highest change score of all the bins. This indicates the greatest amount of change from the beginning of therapy occurs at the end of therapy, which is the desired outcome of therapy. Investigating individual features suggest that this holds true for several features as well, including all values of S for daytime steps (the highest number of steps taken during the last bin) and sedentary percent (the percent of day sedentary is lowest during the last bin). Secondly, on the change score curves, there appears to be at least one trough in between $B_{1}$ and $B_{S}$. More specifically, for S=4, the DDS and VC curves exhibit a $B_{1}\Delta B_{3}$ score that is lower than $B_{1}\Delta B_{2}$ and $B_{1}\Delta B_{4}$. For S=7, there are dips around $B_{1}\Delta B_{4}$ (sw-PCAR, DDS) and $B_{1}\Delta B_{5}$ (VC), while there are dips around $B_{1}\Delta B_{6}$ (sw-PCAR, VC), $B_{1}\Delta B_{7}$ (sw-PCAR, DDS), and $B_{1}\Delta B_{8}$ (VC). The source of these dips is evident in several features, suggesting patients experience a decrease in PA duration (daytime steps decreases, sedentary percent increases), frequency (THR and EHR number of bouts decreases), and intensity (bout steps max decreases, maximum observed HR decreases). In general, the dips occur near, or shortly after, mid-stay of inpatient rehabilitation. A similar trend was observed by the SIRRACT study, which used gait parameters derived from wearable sensors to provide feedback on daily steps, bouts, and walking speed. In the SIRRACT study, 30% of participants decreased their total daily walking time over the course of inpatient rehabilitation [5]. In the current study, we were silent on whether therapists/patients pay attention to the step data. Consequently, our findings represent more ecological circumstances in which we were observing behavior but not necessarily trying to influence behavior with the wearables.

Comparing the REHAB change scores to the average CONTROL change scores (Table 3 and green line in Figure 3a–i), there does not appear to be a consistent relationship that holds for all values of S and algorithms. Upon inspection of Table 3, the average change for REHAB is sometimes lower than the average change for CONTROL, as demonstrated by DDS (S=4,7) and sw-PCAR (S=4,10); however, for all S values of VC, REHAB averages are higher than CONTROL averages. Looking at Figure 3a–i, $B_{1}\Delta B_{S}$ exceeds the CONTROL average, suggesting change during the final bin is more than the typical daily variation, with the exception of DDS (S=4). While the CONTROL group in this study provides some reference information, the high change score values for DDS and sw-PCAR emphasize the importance of age-matching the CONTROL group in future work.

Additional REHAB observations can be made by looking at how each algorithm captures change. sw-PCAR does not leverage any features and, instead, computes change scores directly from the distribution of the data. As the patients’ progress through therapy, the sw-PCAR change scores demonstrate increased variability. The group SD is as low as σ=0.134 for S=4 and as high as σ=0.419 for S=10. All comparisons to the baseline bin generate scores higher than the significance threshold and only bins in the latter half of therapy surpass the CONTROL sw-PCAR average. More representative sw-PCAR significance thresholds could be computed by replacing the value 1.5 in the box plot-based outlier detection method with a parameter, *C*, which is fit based on the dataset, or by exploring other methods to test membership of an observation (i.e., CS) to a sample distribution (i.e., V). If the sample is normal, statistical tests such as Grubb’s test for outliers [37] can be applied. However, the assumption of normality does not hold for all samples of human behavior data. In future work, we plan to investigate data-driven approaches to tuning the parameter C and to investigate data mining techniques relevant to outlier detection.

Like sw-PCAR, all DDS change scores all greater than 0.25. The threshold value of 0.25 was selected based on its prevalence in a previous smarthome study [15] and is perhaps too low for the REHAB group used in the current study. A more appropriate threshold to compare with is the CONTROL average, for which DDS does exceed twice for S=4,7.

Lastly, for the VC approach, there is only one significant result which is $B_{1}\Delta B_{4}$ for S=4. The associated decision tree for this comparison (see Figure 4) finds the features of standard deviation of bouts minutes, moderately/highly active percent, number of THR bouts, and maximum observed heart rate to be the most discriminative (in that order). The tree characterizes the baseline bin, $B_{1}$, as having lower variability in minutes per bout, lower active percent, lower number of exercise bouts, and lower heart rate. $B_{1}\Delta B_{7}$ for S=7 comes quite close (CS=0.667, sig=0.682), while $B_{1}\Delta B_{10}$ for S=10 is quite high, it is not significant because the threshold becomes quite high with a small sample size of N=5 (CS=0.7, sig=0.800). The CONTROL average VC accuracies are quite close to what would be expected for random data and therefore do not provide insight into a potential relationship between the REHAB and CONTROL group. This result is likely due to the small sample size of the CONTROL group.

### 5.3. REHAB Case Studies

Participant 008 had the shortest length of stay (FDC = 4) of the REHAB group and, thus, dictated the value of our minimum split, S=4. Participant 008 was undergoing inpatient rehabilitation to recover from a stroke; participant 008’s DDS scores follow the same trend as for the group with a dip at $B_{1}\Delta B_{3}$, though participant 008’s scores are more pronounced than the group average. On the other hand, participant 008’s sw-PCAR $B_{1}\Delta B_{3}$ and $B_{1}\Delta B_{4}$ scores show a decrease in change from $B_{1}\Delta B_{2}$, which is opposite of the group. Though sw-PCAR does not utilize features to compute its change score, we can investigate the features to provide a context for the detected change. At $B_{1}$, participant 008 took 5954 steps during the daytime, which is the highest step count of participant 008’s data collection period. At $B_{2}$, her daytime steps fell to 3177 (−46.64%). At $B_{3}$, her daytime steps fell to 3520 (−40.88%) and lastly, at $B_{4}$, her daytime steps fell to 4389 (−26.28%). In the case of daytime steps, the greatest change occurs at $B_{1}\Delta B_{2}$, which is a negative change. The remaining comparisons, though still negative, are actually less change from $B_{1}$, but still an improvement.

Participant 014 had the longest length of stay (FDC = 23) of the REHAB group. Participant 014 was undergoing inpatient rehabilitation to recover from a stroke. sw-PCAR and DDS scores for participant 014 both exhibit lower scores at the beginning of data collection and higher scores toward the end. During the last four days of her stay, an impressive change in her daytime steps was noted ($D_{1}$ to $D_{19}$ μ = 1935.42, $D_{20}$ to $D_{23}$ μ = 3898.75). The day before discharge, she achieved a walking bout of 666 steps, the highest out of her data collection period. Interestingly, between days $D_{13}$ to $D_{16}$, participant 014 shows high change relative to previous days and the subsequent four days. From $D_{13}$ onwards, participant 014 exhibits significant change up to her discharge date. Upon investigating the feature changes around this time, on $D_{13}$ participant 014 walks a high of 495 steps in a single bout (compared to 173 steps on $D_{12}$) and on $D_{15}$ participant 014 achieves her only bout in the THR zone during the entire length of data collection. While we can describe participant 014’s change in terms of PA features, we do not have context to explain why she exerted more effort during some periods over others. Aligning PACD results with notes from therapists or family members describing the participant’s mental, emotional, and physical state is an insightful direction for future work.

## 6. Conclusions

In this paper, we analyze longitudinal physical activity data collected from inpatient rehabilitation participants (REHAB group) and healthy control subjects (CONTROL group) to gain insights into changes over time. To detect changes over time, we utilize the Physical Activity Change Detection (PACD) change analysis approach. We expanded PACD to detect changes on a daily basis and to summarize changes across participants with varying lengths of data collection. Our approach is also effective for comparing physical activity across demographic groups. The results indicate that our methods are able to effectively capture significant changes in the physical activity and heart rate data for both the REHAB and CONTROL groups. Specifically, we conclude that the greatest amount of change occurs at the end of inpatient rehabilitation, the amount of change over time does not necessarily increase linearly, and when compared to the CONTROL group, the REHAB group exhibits greater change. This suggests that the REHAB group is improving their physical activity and not just experiencing normal variations in walking that are present in the CONTROL group. Lastly, the aspects of physical activity showing the greatest change for the REHAB group include daily steps, number of minutes in a bout, variability of the number of minutes in a bout, maximum number of steps in a bout, and maximum heart rate.

Among the limitations of this work was the absence of age-matching among the CONTROL group and the REHAB group. With an age-matched control group, the normative change score values would be more clinically relevant for comparison with the changes exhibited by the REHAB group. Another limitation includes the small sample size of our REHAB group (N = 15). Consequently, future work includes increasing the REHAB sample size, collecting an additional dataset of age-matched controls who continuously wear fitness devices with heart rate sensors, and collecting ground truth physical activity data for comparison. Another direction for future work involves designing a data-driven change significance test for DDS, adding parameter tuning for the current sw-PCAR test, and performing additional research regarding the accuracy of each change score algorithm. We also plan to investigate providing real-time PACD data as intentional feedback to allow therapists to tailor therapeutic activity to achieve activity goals. In summary, the data analysis approach and results we presented are valuable for the pervasive computing community because we provide evidence that passively-collected data from commercially-available, wearable devices can be analyzed using PACD to gain highly useful insights for inpatient rehabilitation. 

## Figures and Tables

**Table 1:** REHAB group participant demographics.

ID	Sex	Age	RIC	Pre-Hospital IPAQ Category	FDC	DBC	FIM_A Motor_	FIM_D Motor_	Beta-Blockers?
001	M	75	Orthopedic Disorder	High	7	2	56	83	No
002	F	46	Stroke	High	10	2	54	85	No
003	F	74	Pulmonary Disorder	Low	16	2	35	79	No
004	M	48	Cardiac Disorder	Low	18	2	59	84	No
005	M	55	Debility	High	7	3	50	69	Yes
006	M	59	Medically Complex Condition	Moderate	11	2	51	84	Yes
007	M	68	Stroke	High	9	1	55	84	No
008	F	50	Stroke	Moderate	4	3	56	86	No
009	F	80	Lower Extremity Fracture	High	8	1	44	59	No
010	F	60	Stroke	High	7	3	37	63	No
011	F	74	Stroke	High	5	2	49	70	No
012	M	61	Stroke	Low	9	1	60	83	Yes
013	M	78	Cardiac Disorder	Low	5	2	53	86	Yes
014	F	48	Stroke	High	25	2	37	75	No
015	M	44	Stroke	Moderate	5	1	56	81	No
Mean		61.33			9.73	1.93	50.13	78.07	
SD		12.66			5.79	0.70	8.19	8.80	

A = admission, D = discharge, DBC = days before data collection began, F = female, FDC = full days of data collection, FIM = Functional Independence Measure, M = male, SD = standard deviation.

**Table 2:** Features extracted from step and heart rate time series data.

Time Series	Feature	Description
Steps	Total steps	Total steps per period: (1) daytime, (2) nighttime
	Number of bouts	Count of detected step-based PA bouts during daytime
	Max steps per bout	Highest number of steps taken in a bout during daytime
	Minutes per bout	Mean and SD of duration of bouts during daytime
	PA intensity percentage	Percentage of 24-h day that is (1) sedentary (<5 steps/min), (2) low (5 ≤ steps/min < 40), (3) moderate (40 ≤ steps/min < 100), (4) high (≥100 steps/min), (5) active (≥5 steps/min) intensity levels during daytime
	Daily activity ratio	Number of daytime active minutes divided number of nighttime active minutes
Heart rate	Maximum heart rate	Maximum heart rate during daytime
	Zone number of bouts	Count of detected bouts in (1) THR zone and (2) EHR zone during daytime
	Zone minutes per bout	Mean, SD, total, and maximum of duration of bouts in (1) THR zone and (2) EHR zone during daytime

EHR = excessive heart rate, PA = physical activity, SD = standard deviation, THR = target heart rate.

**Table 3:** Summary of change scores (CS) for each group (CONTROL and REHAB).

		CONTROL			REHAB		
		S=4	S=7	S=10	S=4	S=7	S=10
sw-PCAR	Mean	0.391	0.403	0.715	0.278	0.404	0.600
	SD	0.157	0.166	0.382	0.134	0.230	0.419
DDS	Mean	0.457	0.434	0.449	0.398	0.423	0.456
	SD	0.072	0.049	0.040	0.029	0.040	0.077
VC	Mean	0.433	0.467	0.506	0.511	0.478	0.556
	SD	0.076	0.140	0.174	0.107	0.150	0.101
	Significance threshold	0.667	0.667	0.667	0.633	0.682	0.800

SD = standard deviation.

**Table 4:** Daytime steps for individual REHAB participants (S=4).

Participant	$B_{1}$	$B_{2}$	$B_{1}\Delta B_{2}$	$B_{3}$	$B_{1}\Delta B_{3}$	$B_{4}$	$B_{1}\Delta B_{4}$
001	2184.00	3728.50	70.72	2230.50	2.13	3382.00	54.85
002	2008.67	1404.33	−30.09	2351.00	17.04	3232.00	60.90
003	3081.25	3647.00	18.36	4063.75	31.89	4187.25	35.89
004	2615.40	1999.80	−23.54	4188.50	60.15	4325.75	65.40
005	227.00	652.50	187.44	942.00	314.98	1936.00	752.86
006	500.33	2144.00	328.51	1493.67	198.53	2532.00	406.06
007	4466.33	2768.50	−38.01	3056.00	−31.58	4919.00	10.14
008	5954.00	3177.00	−46.64	3520.00	−40.88	4389.00	−26.28
009	594.50	1178.50	98.23	531.00	−10.68	681.00	14.55
010	1625.50	1862.00	14.55	1811.50	11.44	1507.00	−7.29
011	866.00	501.00	−42.15	1680.00	94.00	708.00	−18.24
012	1218.67	1959.00	60.75	1637.00	34.33	1859.00	52.54
013	2487.00	3246.00	30.52	1375.00	−44.71	1608.00	−35.34
014	2103.17	1854.17	−11.84	1728.33	−17.82	3650.80	73.59
015	4006.50	1647.00	−58.89	3491.00	−12.87	3074.00	−23.27
Mean	2262.55	2117.95	37.20	2273.28	40.40	2799.39	94.42
SD	1602.30	1010.65	104.40	1134.56	98.01	1369.43	210.71

SD = standard deviation.

**Table 5:** Number of THR zone bouts for individual REHAB participants (S=4).

Participant	$B_{1}$	$B_{2}$	$B_{1}\Delta B_{2}$	$B_{3}$	$B_{1}\Delta B_{3}$	$B_{4}$	$B_{1}\Delta B_{4}$
001	5.50	5.50	0.00	6.50	18.18	7.00	27.27
002	0.00	0.33	N/A	3.00	N/A	6.00	N/A
003	8.25	16.75	103.03	13.75	66.67	14.50	75.76
004	0.80	2.60	225.00	4.25	431.25	6.00	650.00
005	6.50	11.50	76.92	7.00	7.69	9.00	38.46
006	9.33	11.00	17.86	9.00	−3.57	14.50	55.36
007	2.00	0.00	−100.00	0.50	−75.00	1.00	−50.00
008	1.00	0.00	−100.00	1.00	0.00	0.00	−100.00
009	5.50	6.00	9.09	6.50	18.18	5.00	−9.09
010	0.00	0.00	N/A	0.00	N/A	0.00	N/A
011	15.00	17.00	13.33	10.00	−33.33	14.00	−6.67
012	8.00	9.50	18.75	8.00	0.00	10.00	25.00
013	13.00	7.00	−46.15	15.00	15.38	16.00	23.08
014	0.00	0.00	N/A	0.17	N/A	0.00	N/A
015	0.00	0.00	N/A	0.00	N/A	0.00	N/A
Mean	4.99	5.81	19.80	5.64	40.50	6.87	66.29
SD	4.98	6.14	92.68	4.95	134.20	5.94	199.74

SD = standard deviation.
